# Developing a Novel Integrated Generalised Data Envelopment Analysis (DEA) to Evaluate Hospitals Providing Stroke Care Services

**DOI:** 10.3390/bioengineering8120207

**Published:** 2021-12-10

**Authors:** Mirpouya Mirmozaffari, Elham Shadkam, Seyed Mohammad Khalili, Maziar Yazdani

**Affiliations:** 1 Department of Industrial Engineering, Dalhousie University, 5269 Morris Street, Halifax, NS B3H 4R2, Canada; 2 Department of Industrial Engineering, Faculty of Engineering, Khayyam University, Mashhad 9189747178, Iran; e.shadkam@khayyam.ac.ir (E.S.); m.khalili@khayyam.ac.ir (S.M.K.); 3 School of Built Environment, University of New South Wales, Sydney 2052, Australia; Maziar.yazdani@unsw.edu.au

**Keywords:** generalised data envelopment analysis, multiple objective linear programming, interactive approach, stem method, stroke, health, hospital

## Abstract

Stroke is the biggest cause of adult disability and the third biggest cause of death in the US. Stroke is a medical emergency, and the treatment given in the early hours is important in shaping the patient’s long-term recovery and prognosis. Despite the fact that substantial attention has been dedicated to this complex and difficult issue in healthcare, novel strategies such as operation research-based approaches have hardly been used to deal with the difficult challenges associated with stroke. This study proposes a novel approach with data envelopment analysis (DEA) and multi-objective linear programming (MOLP) in hospitals that provide stroke care services to select the most efficient approach, which will be a new experiment in literature perception. DEA and MOLP are widely used for performance evaluation and efficiency measurement. Despite their similarities and common concepts, the two disciplines have evolved separately. The generalised DEA (GDEA) cannot incorporate the preferences of decision-makers (DMs) preferences and historical efficiency data. In contrast, MOLP can incorporate the DM’s preferences into the decision-making process. We transform the GDEA model into MOLP through the max-ordering approach to (i) solve the problem interactively; (ii) use the step method (STEM) and consider DM’s preferences; (iii) eliminate the need for predetermined preference information; and (iv) apply the most preferred solution (MPS) to identify the most efficient approach. A case study of hospitals that provide stroke care services is taken as an example to illustrate the potential application of the proposed approach method.

## 1. Introduction and Background

Stroke is a serious public health issue, impacting more than 10 million people worldwide each year [1]. Stroke is the world’s second biggest cause of death, accounting for around 5.5 million deaths per year [2]. The burden of stroke lies not only in its high mortality rate, but also in the significant morbidity it causes, which results in up to half of survivors being chronically disabled [2]. One-quarter of stroke victims die within a month of the onset of the disease [1]. Stroke accounts for more than 4% of direct healthcare costs in developed nations [3]. Each year, stroke imposes considerable costs to societies and billions of dollars are spent on stroke treatment and care. For example, stroke-related costs in the United States came to nearly $46 billion between 2014 and 2015 [4]. Annually, the US experiences an estimated 610,000 new cases of stroke [4]. Approximately 80% of all strokes are ischemic, meaning they are caused by a stoppage of blood flow to the brain [3].

According to the findings of the Saver [5] model, the average patient loses 1.9 million neurons every minute a stroke goes untreated. Therefore, every second is important for patients suffering from acute ischemic stroke, as well as for the doctors and health staff who are caring and treating them. Although wide availability of treatment to appropriate patients remains a major concern for a number of health systems worldwide [6], at the moment, only about 5% of stroke sufferers receive treatment, resulting in disability prevention in only six patients per 1000 ischemic strokes [3]. Administration rates, even in some developed countries such as Australia, in particular, remain low [6]. This might be related to the relatively small therapeutic time window and a scarcity of doctors who specialise in acute stroke therapy across the world.

Much effort has been expended in recent years to comprehend and apply techniques to improve services provided by hospitals [7], particularly for stroke patients. Even though there is a clear potential for Operations Research (OR) to assist health decision-makers [8], as evidenced by numerous successful OR applications to the other domains, there is a clear research gap in employing OR techniques in providing more reliable services for stroke patients. Operation Research is a field of study that uses advanced analytical methods to understand complex systems and make the right decisions [9,10,11]. Simulation techniques [12,13,14,15], mathematical optimisation [16,17], and decision analysis [18] are just a few of the ways OR helps enterprises and organisations improve their operations. OR, with its emphasis on increasing efficiency [19,20], cost-effectiveness [21,22], and decision-making [23,24], is particularly valuable for analysing complicated health issues, particularly in contexts where disease burden is high, but health systems are not well prepared. The purpose of this study is to fill a research gap and add to the health and OR literature by showing how OR techniques, such as DEA, can be utilised to support decisions in hospitals that provide stroke care services.

The remainder of the paper is organised as follows. Section 2 presents the background of the research. In Section 3, the necessity and importance of the proposed method, research gaps, contributions and flowchart are presented. Section 4 provides a brief definition of the GDEA. In Section 5, the dual model of the GDEA model is presented. Section 6 presents the relationship between GDEA and MOLP. Section 7 demonstrates the proposed interactive method and discusses the three steps of research methodology (Step 1—normalisation of data, Step 2—Evaluation of efficiency, Step 3—Attainment to MPS). Section 8 establishes a brief description of the STEM method. In Section 9, the application of the proposed approach in three proposed steps of Section 7 in the real world in hospitals that provide stroke care services is examined. Finally, the conclusion and future works of the experimental consequences are discussed in the last section.

## 2. Background

DEA is a non-parametric method proposed by Charnes et al. [25] to measure the efficiency of decision-making units (DMUs) and estimate production frontiers, and its variants have been widely used in different domains [26,27,28,29]. While Charnes and Cooper [30] made significant contributions to expanding MOLP methods, they did not devote much attention to bridging the gap between these two fields. Despite different terms used in DEA and MOLP, these two fields are structurally similar. Jahanshahloo and Foroughi [31], Korhonen and Syrjänen [32], and Li and Reeves [33] stated that DEA is a multi-criteria decision analysis (MCDA) tool that takes into consideration multiple inputs and outputs. Quariguasi Frota Neto and Angulo-Meza [34] have shown the common elements and overlap between these two fields.

Several studies have developed integrated DEA and MOLP frameworks such as (Belton and Vickers [35]; Belton and Stewart [36]; Joro et al. [37]; Stewart [38]; Estellita Lins et al. [39]; Hong and Jeong [40]. Belton and Vickers [35] proposed an integrated DEA and multi-attribute value function approach. They implemented the proposed approach as a visual interactive decision support system and overcame some of the limitations of DEA. Belton and Stewart [36] suggested that the DEA and MCDA models can be used complementarily. Joro, Korhonen and Wallenius [37] compared DEA with a reference direction MOLP method to identify efficient units. Stewart [38] compared Pareto optimal and efficiency concepts in DEA and MCDA and connected the ratio efficiency over DEA, and measured distance in the input and output space based on linear value functions. Estellita Lins, Angulo-Meza and Moreira Da Silva [39] proposed a multi-objective approach that considers each objective as the basis for a posterior priority through separate predictions of each variable (input or output), thus allowing one to achieve a goal at every extreme-efficient point on the Pareto frontier. Hong and Jeong [40] proposed an innovative approach to finding efficient facility location-allocation schemes using an integrated DEA and multi-objective programming method. They showed while DEA and MOLP cannot substitute each other, they can complement each other. They also demonstrated that MOLP is a particular extension of the DEA model, and DEA provides opportunities for new application areas to MOLP models. Azadeh et al. [41] proposed a new approach for maintenance policy and planning problems by combining DEA and simulation for learning. Azadeh et al. [42] presented an integrated fuzzy or simulation–fuzzy DEA to cope with a maintenance activity planning problem. Several outputs, including machines and operators’ availability, reliability, efficiency, and queue length, were considered in the integrated framework.

The GDEA model has been used in various studies to optimise multi-objective problems. Shadkam and Bijari [43] used the GDEA model in a simulation-optimisation supplier selection problem. Using the GDEA model, the multi-objective problem has become a single-objective problem. Yun and Nakayama [44] presented an integrated method for producing efficient frontiers in multi-objective optimisation problems based on the genetic algorithm and GDEA techniques. The proposed approach can effectively identify efficient frontiers for concave and convex objective functions. Jahanshahloo and Foroughi [31] presented a method combining DEA concepts and a multi-objective model to search for a composite alternative and assess the units’ efficiency. They also investigated the relationship between some DEA and multi-objective models. Tavana et al. [45] established an equivalence relationship between MOLP problems and combined-oriented DEA models using a direction distance function designed to account for desirable and undesirable inputs and outputs together with uncontrollable variables.

Allen et al. [46] defined value judgments to reflect DM’s priorities in the process of evaluating efficiency. Various methods (i.e., limiting composite outputs and inputs and weight ratios and proportions) have been developed to consider the DM’s preferences in DEA [47], including the target and goal setting models of Golany et al. [48], declaration area [49], weight restriction approach [50], imposing limits on specific weights [51], and relation perception, by setting the related output-input stages to obtain the values which fit into a specified case [52,53]. Zhu [54] integrated performance information with the modified DEA model, while Golany, Roll and Rybak [48] applied hypothetical DMUs to provide priority.

However, all the mentioned methods need to predetermine the DM’s preference information, which can be problematic or impossible and often impractical in a real-world problem. Many decision-making problems can be modelled as multi-objective problems, and finally, several Pareto solutions are offered as candidates to the DM [55]. For DM’s, selecting MPS from the several Pareto solutions is a critical problem. Interactive multi-objective problems proposed many solutions to solve this issue [56,57,58,59]. These methods provide the most preferred explanation by implementing the two iterative phases:

(a). Moving the optimisation part, Pareto frontier, close to the specified aspiration level.
(b). Modifying the aspiration level according to the trade-off method.

The most interesting method in terms of the DM’s preference, without setting targets and with no predetermined preference information, is implementing an interactive approach by applying two suggested optimisation methods. Golany [60] presented a collaborative method using both techniques. Post and Spronk [61] integrated interactive goal programming and DEA. Information is received in the form of the upper and lower bounds of the inputs and outputs. Then, Joro, Korhonen and Wallenius [37] demonstrated that combining DEA and MOLP creates synergy, and the DEA is related to MOLP methods. In addition, value efficiency analysis (VEA) considered the DM’s preference effectively in DEA. Thus, it is not surprising that in recent years, many scientists have focused on developing efficient algorithms for solving multi-objective mixed-integer linear programs, such as Halme et al. [62], Charkhgard et al. [63], Elliot et al. [64].

Halme, Joro, Korhonen, Salo and Wallenius [62], Korhonen et al. [65], Joro et al. [66], and Yang et al. [67] studied equivalent approaches between DEA and MOLP such as the two suggested approaches, namely the super-ideal point model and the shortest distance model, respectively. Additionally, in research by Wong et al. [68], the GDEA model was selected due to its flexibility for evaluating the units’ efficiency. The GDEA model can create the basic DEA model, such as FDH, CCR, and BCC models, in a unified structure. Yun et al. [69] have shown that the GDEA model evaluates the efficiency of units considering DM’s preferences. While [70] proposed an aspiration level method based on the GDEA model for considering DM’s preferences in the decision-making process. Their interactive approach determines some limited Pareto frontier ranges based on DM’s preferences, and DMs select MPS among the proposed set of the Pareto frontier. Their method did not provide MPS, which is the main target of all the interactive techniques. Therefore, that was the main weakness of their method. Additionally, the mentioned approach requires predetermined preference information, which is not determined in many decision-making problems.

Lotfi et al. [71], and Lotfi et al. [72] presented the relationship between MOLP and the DEA model and showed how DEA could be considered interactive for the MOLP model. They used Zionts-Wallenius (Z-W) [73] method to reflect the DM’s preferences to the solving process.

The presented approach in this study is similar to the method of Lotfi, Jahanshahloo, Soltanifar, Ebrahimnejad and Mansourzadeh [72] in some aspects. They developed the DEA method interactively, while this study presents the interactive approach of the GDEA method, which is a more comprehensive and practical model. While their model used the min-ordering approach to convert the DEA dual model to MOLP, the current study uses the max-ordering approach to convert the GDEA dual model to MOLP. Furthermore, Z-W was used in previous studies to consider DM preferences in the multi-objective model, while this research employs the STEM method.

## 3. Research Gaps, Contributions, and Flowchart

The literature review techniques necessitate prior knowledge of DM preferences, which is often complex and challenging to obtain. In manufacturing or service organisations and management problems, decision-making can be more difficult and often uncertain due to multiple characteristics and conflicting objects. Multi-objective programming methods such as MOLP can be used to solve such problems. Such methods do not require predetermined information, and DM preferences can be applied while solving the problem. Therefore, the main advantage of the proposed approach compared to other approaches is that it does not require prior knowledge of DM. In the main structure of the GDEA model, DM preferences cannot be considered in the efficiency calculation, and the results may not be acceptable by the DM.

The proposed approach has the advantage of making several basic DEA models available due to the use of GDEA. Due to the interactive structure of the proposed approach, decision-maker preferences can be applied to the final efficiency results, which leads to better acceptance of the results by the DM. However, in the main structure of DEA and GDEA, there is no structure to consider decision-makers preferences. The superiority of the proposed approach compared to other approaches is described as qualitative and not quantitative. As mentioned earlier, the proposed method does not require prior DM preferences. It finally provides the MPS at the end of the problem. The process is simple, which is present in the case study. Because the dual form of GDEA is structurally (in terms of objectives and constraints) similar and equivalent to the multi-objective mathematical model, this way, the outputs of the DMU in GDEA can be converted into objectives of a multi-objective mathematical model. The STEM method in interactive DEA has not been used so far, and this is the first time it has been used. The STEM method is an interactive approach for multi-objective problems and not for DEA models, but in this paper, we have been able to use the interactive nature of the STEM approach by converting DEA to multi-objective. In conclusion, the main contributions of this study can be summarised as follows:

- This study presents an interactive GDEA model approach that overcomes the drawbacks of the previous approaches. The proposed interactive method is less complex than previous methods and does not require predetermined preference information.
- This paper presents an interactive GDEA model that employs the max-ordering approach and the STEM method. In other words, a relationship is established between the GDEA dual model and the MOLP problem, and it is demonstrated how a GDEA model can be evaluated interactively by converting to the MOLP problem using the max-ordering approach. The problem is then solved using an interactive approach based on MOLP. The STEM approach is used to consider the DM’s preferences in the decision-making process.
- While OR approaches can benefit the healthcare sector, there is a significant research gap in using OR tools such as DEA and MOLP in challenging healthcare issues, particularly in evaluating stroke care services. Furthermore, while the benefits of appropriate care services of stroke care patients have been highlighted in the literature, there is a limitation of using OR models to demonstrate the long-term benefits of more immediate access to various stroke care involvements on patients’ lifetime outcomes. As a result, one of the primary purposes of this study is to fill a critical research gap.
- A case study is used to evaluate the proposed approach. The results show that the proposed approach contributes new theoretical and practical insights to a growing body of knowledge about hospital strategies and implications for hospital planners, managers, and policymakers in countries where health centres are increasingly facing challenging issues, particularly in providing reliable services for stroke care.

**Figure 1:**
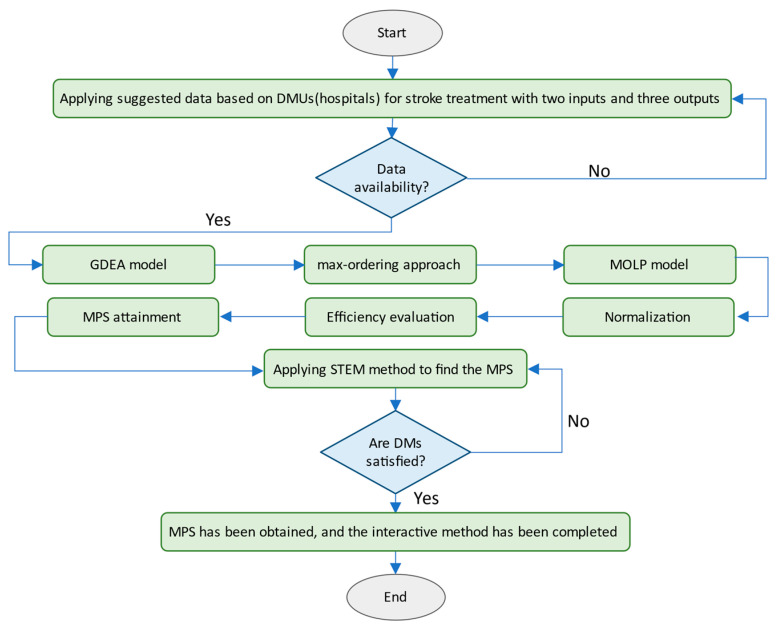
Flowchart of the proposed interactive model.

Figure 1 shows the flowchart of the proposed model. The GDEA model is transformed into MOLP through the max-ordering approach or summarising the proposed method into normalisation, efficiency evaluation and MPS attainment before applying STEM. After applying the suggested approach, the best option will be selected. In a nutshell: (i) solve the problem interactively; (ii) use the step method (STEM) and consider DM’s preferences; (iii) eliminate the need for predetermined preference information; and (iv) apply the MPS to identify the most efficient approach.

## 4. Generalised Data Envelopment Analysis (GDEA)

**Figure 2:**
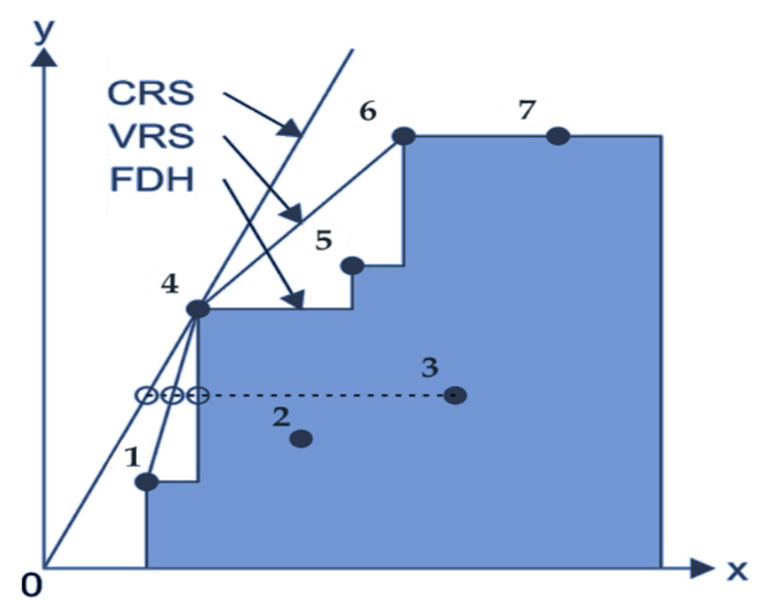
Relationship between the CRS, VRS and FDH models. Reprinted from Ref. [74].

Several models have been developed to estimate the relative efficiency of decision-making units (DMUs) under different assumptions, including the Charnes, Cooper and Rhodes (CCR) model or constants returns-to-scale (CRS), the Banker, Charnes and Cooper (BCC) model or variable returns-to-scale (VRS), the free disposal hull (FDH) model. These models are characterised by how the production possibility set and the dominating structure are identified. The production possibility set of the FDH model is accomplished by evaluating the FDH model inversely based on the CCR and BCC models. Figure 2 shows the relationship between the CRS, VRS and FDH models.

**Figure 3:**
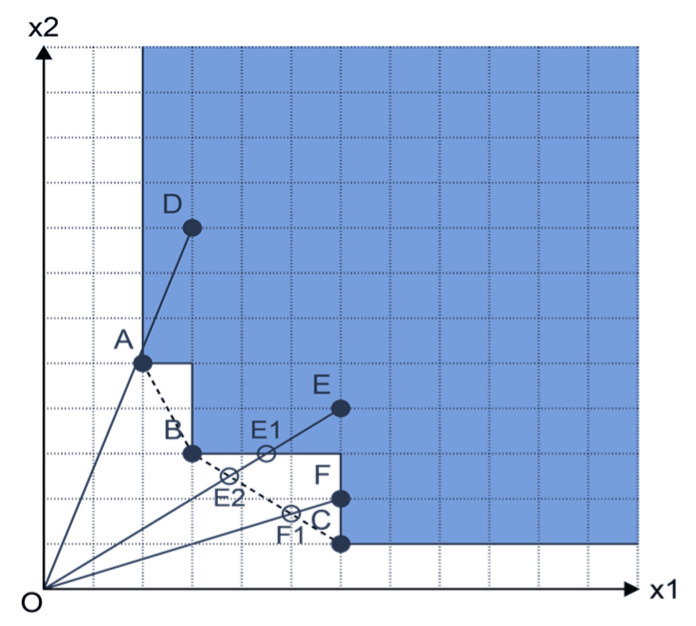
The efficiency comparison of CCR, BCC and FDH models. Reprinted from Ref. [74].

The FDH model provides the free impossibility of creating the production possibility set. Therefore, the frontier line for the FDH is established based on the detected inputs and outputs. Figure 3 shows the production possibility set in FDH. There are two inputs and one output for six DMUs, categorised A to F. Based on the suggested evaluation, the efficiency score of the FDH model is between 0 and 1. Therefore, in the input-oriented approach, the efficiency for the FDH input-oriented model is permanently more than the input-oriented VRS and CRS models.

For the BCC model, the efficient units are A, B and C (the efficiency score of E for the BCC model is $\theta_{E\cdot BCC\cdot input-oriented}=OE_{2}/OE\;$). However, in the FDH model, the efficient units are A, B, C and F (the efficiency score of E for the FDH model is $\theta_{E\cdot FDH\cdot input-oriented}=OE_{1}/OE$). Thus, the efficiency score of FDH is more than that of BCC, and the efficiency score of BCC is more than that of CCR. The present study employs the GDEA model, which presents the models mentioned above in a unified structure. This model includes a parameter, α, which plays a key role in presenting several basic DEA methods. The following GDEA method assumes that there are *n* DMUs (${\mathrm{DMU}}_{j}:j=1,\ldots n)$ to be investigated where ${\mathrm{DMU}}_{j}$ uses m inputs $x_{j}=\left(x_{1j},\ldots ,x_{mj}\right)$ to produce s outputs $y_{j}=\left(y_{1j},\ldots ,y_{sj}\right)$.

(1) $$\mathrm{Max}\;\Delta_{o}$$

s.t.

(2) $$\Delta_{o}\leq {\tilde{d}}_{j}+\alpha \left(\overset{s}{\underset{r=1}{\sum }}u_{r}\left(y_{ro}-y_{rj}\right)+\overset{m}{\underset{i=1}{\sum }}v_{i}\left(-x_{io}+x_{ij}\right)\right)\;,\;\;\;j=1,\ldots ,n$$

(3) $$\overset{s}{\underset{r=1}{\sum }}u_{r}+\overset{m}{\underset{i=1}{\sum }}\nu_{i}=1$$

(4) $$u_{r}\;\geq \varepsilon \;,\;\;r=1,\ldots ,s\;\;\;\;\;$$

(5) $$v_{i}\geq \varepsilon \;,\;\;\;i=1,\ldots ,m\;\;\;\;$$

where parameter α>0  is proportionally specified according to the specific DEA model, $\mu_{j}\;\mathrm{and}\;\nu_{i}$ are the weights of outputs and inputs, respectively, ε is a non-Archimedean infinitesimal value which is imposed to the model for preventing the weights from taking zero values (see Amin and Toloo [75] and Toloo et al. [76]), and $\Delta_{o}$ is the efficiency score of ${\mathrm{DMU}}_{o\in \left\{1,\ldots ,n\right\}}$ and

(6) $${\tilde{d}}_{j}=\{\begin{array}{cc} \left(y_{po}-y_{pj}\right)u_{p} & \mathrm{if}\;y_{po}-y_{pj}=\max\left\{y_{ro}-y_{rj},x_{ij}-x_{io}:\forall r,\;\forall i\right\}\;\\ \left(x_{qj}-x_{qo}\right)v_{q} & \mathrm{if}\;x_{qj}-x_{qo}=\max\left\{y_{ro}-y_{rj},x_{ij}-x_{io}:\forall r,\;\forall i\right\} \end{array}$$

For example, if $(y_{1o}-y_{1j},\;x_{1j}-x_{1o})=\left(2,-1\right)$, then ${\tilde{d}}_{j}=2u_{1}$. Note that the possible ties can be broken arbitrarily. Clearly, from the first set of constraints for j=o we obtain ${\tilde{d}}_{o}=0$ and $\Delta_{o}\leq 0$. Moreover, by summing up the weight restrictions $u_{r}\geq \varepsilon$ and $v_{i}\geq \varepsilon$ for r=1,…,s and i=1,…,m, respectively, and considering the constraint $\sum_{r=1}^{s}u_{r}+\sum_{i=1}^{m}\nu_{i}=1$, it is easy to know that $\left(m+s\right)\varepsilon \leq \sum_{r=1}^{s}u_{r}+\sum_{i=1}^{m}\nu_{i}=1$ or equivalently $\varepsilon \leq \frac{1}{m+s}$ which provides an upper bound for the non-Archimedean epsilon. For a given α, DMU*_o_* is α-efficient if and only if $\Delta_{o}=0,$ and otherwise, it is α-inefficient. The DMU*_o_* is FDH-efficient if and only if $\Delta_{o}=0$ for sufficiently small and positive α.

- The DMU*_o_* is BCC-efficient if and only if $\Delta_{o}=0$ for sufficiently large and positive α.
- The DMU*_o_* is CCR-efficient if and only if $\Delta_{o}=0$ for sufficiently large and positive α and extra constraint $\sum_{r=1}^{s}u_{r}y_{ro}-\sum_{i=1}^{m}\nu_{i}x_{io}=0$ should be added to the model (1).

It is not possible to know in advance how small/large enough is small/large, and therefore the value of an experiment is given. The most important thing is to find how the efficiency of each DMU changes as the value of α changes. For more information about the GDEA method, refer to [69]. Therefore, in general, the following states are established:

- If α is sufficiently small and positive, then Equations (1)–(5) turn to the FDH model;
- If α is sufficiently large and positive, then Equations (1)–(5) turn to the BCC model;
- If α is a sufficiently large and positive and extra constraint $\sum_{r=1}^{s}u_{r}y_{ro}-\sum_{i=1}^{m}\nu_{i}x_{io}=0$ is added to Equations (1)–(5), then the model turns to the CCR model.

## 5. The Dual Model of the GDEA

Next, we formulated the following dual model of the GDEA model as the basic method for subsequent considerations and denoted it by GDEA_D_:

(7) $$\min\omega_{o}-\varepsilon \left(\overset{s}{\underset{r=1}{\sum }}t_{r}^{y}+\overset{m}{\underset{i=1}{\sum }}t_{i}^{x}\right)$$

s.t.

(8) $$\overset{n}{\underset{j=1}{\sum }}\left\{\alpha \left(y_{ro}-y_{rj}\right)+{\tilde{d}}_{rj}\right\}\;\lambda_{j}-\omega_{o}+t_{r}^{y}+T\cdot D_{r}^{y}=0\;,\;r=1,\ldots ,s$$

(9) $$\overset{n}{\underset{j=1}{\sum }}\left\{\alpha \left(-x_{io}+x_{ij}\right)+{\tilde{d}}_{ij}\right\}\;\lambda_{j}-\omega_{o}+t_{i}^{x}-T\cdot D_{i}^{x}=0\;,\;i=1,\ldots ,m$$

(10) $$\overset{n}{\underset{j=1}{\sum }}\lambda_{j}=1,$$

(11) $$\lambda_{j}\geq 0,\;j=1,\ldots ,n$$

(12) $$t_{r}^{y}\geq 0,\;r=1,\ldots ,s$$

(13) $$t_{i}^{x}\geq 0,\;i=1,\ldots ,m$$

(14) T is free

where $\lambda_{j}$ and $\omega_{o}$ are corresponding dual variables to the first and second constraints sets in the primary GDEA Equations (1)–(5), respectively, $t_{r}^{y}$, and $t_{i}^{x}$ are the slack variables. So, $\omega_{o}-\varepsilon \left(\sum_{r=1}^{s}t_{r}^{y}+\sum_{i=1}^{m}t_{i}^{x}\right)$ and ∆^∗^ are the same in the primal and dual methods. Due to the non-Archimedean property of ε and strong duality of GDEA and GDEA_D_, $\omega_{o}^{*}$ is not greater than zero. The decision variable T is related to extra constraint $\sum_{r=1}^{s}u_{r}y_{ro}-\sum_{i=1}^{m}\nu_{i}x_{io}=0$ in primary GDEA Equations (1)–(5), the coefficients $D_{r}^{y}$ and $D_{i}^{x}$ will be equal to $y_{ro}$ and $x_{io}$ in extra mentioned constraint, respectively. Similar to the primary model of GDEA, ${\tilde{d}}_{ij}$ and ${\tilde{d}}_{rj}$ are the components of the matrix ${\left(-x_{1o}+x_{1j},\ldots ,-x_{io}+x_{ij}\right)}^{T}$ and $\left(y_{1o}-y_{1j},\;\ldots ,y_{ro}-y_{rj}\right),\;\mathrm{respectively}$, replaced by zero, except for the highest component in each column (for example, if $\left(-x_{1,o}+x_{1,5},-x_{2,o}+x_{2,5},-x_{3,o}+x_{3,5}\right)=\left(2,-1,3\right)$ and $\left(y_{1o}-y_{1,5},y_{2o}-y_{2,5}\right)=\left(5,-3\right)$ then ${\tilde{d}}_{ij}={\tilde{d}}_{3,5}=3$ applying for inputs and ${\tilde{d}}_{rj}={\tilde{d}}_{2,5}=5$ applying for outputs). When T=0, the BBC and FDH models developed, but for the CCR model, T≠0 [69]. ω^*^ in the GDEA_D_ model has the same meaning as ∆^∗^ in the GDEA model [77], $\lambda_{j}$ presents the domination relation between DMU*_o_* and other DMUs. The DMU*_o_* is dominated by DMU_s_ if $\lambda_{s}\neq 0$ for some *s*. $t_{r}^{y}$, and $t_{i}^{x}$ indicate the slackness in output and input values in the efficiency appraisal process, correspondingly. Generally, the following cases are established:

**Case** **1.** If α is sufficiently small and positive and T=0, then Equations (7)–(14) turn to the FDH model.
**Case** **2.** If α is sufficiently large and positive and T=0 , then Equations (7)–(14) turn to the BCC model.
**Case** **3.** If α is sufficiently large and positive and T≠0, then Equations (7)–(14) turn to the CCR model.

Let $\left(\omega_{o}^{*}\;,\;\lambda^{*},\;t^{x*},t^{y*},T^{*}\right)$ be the optimal solution for Equations (7)–(14) where $\lambda^{*}=\left(\lambda_{1}^{*},\ldots ,\lambda_{n}^{*}\right)$, $t^{x*}=\left(t_{1}^{x*},\ldots ,t_{s}^{x*}\right)$, and $t^{y*}=\left(t_{1}^{y*},\ldots ,t_{m}^{y*}\right)$. ${\mathrm{DMU}}_{o}$ is α-efficient if and only if the following conditions meet:

(a) $\omega_{o}^{*}=0$
(b) The slack variables are zero, i.e., $t^{x}=0_{s}$ and $t^{y}=0_{m}$ where $0_{s}$ is the origin in ${\mathbb{R}}^{s}$.

Otherwise, it is α-inefficient. Due to the strong duality of (GDEA) and (GDEA_D_), the value of $\omega_{o}$ does not exceed zero. See Yun, Nakayama and Tanino [69] for more information about relationships between GDEA_D_ and basic DEA models.

## 6. The Relationship between GDEA and MOLP

In a DEA model, the efficiency value is evaluated by maximising DMU’s output or minimising DMU’s inputs or outputs, or simultaneously minimising DMU’s inputs and maximising DMU’s output. Therefore, it can be considered a kind of multi-objective problem. This paper studies the relationship between GDEA and the multi-objective program. Suppose a MOLP has *s* objectives, such a model can be written as follows:

(15) $$\min f\left(\lambda \right)=\left[f_{1}\left(\lambda \right),\;\ldots ,\;f_{s}\left(\lambda \right)\right]$$

s.t.

(16) λ∈Ω

where Ω is a feasible space and $f_{r}\left(\lambda \right)\;\left(r=1,\ldots ,\;s\right)$ are continuous objective functions. In a multi-objective problem, it is impossible to find an answer that optimises all objective functions concurrently and represents Pareto solutions. For more information, see [78]. To find non-dominated solutions, the MOLP is based on max-ordering:

(17) $$\min f\left(\lambda \right)=\left[f_{1}\left(\lambda \right),\;\ldots ,\;f_{s}\left(\lambda \right)\right]$$

s.t.

(18) λ∈Ω

The max-ordering model can be written as following model by an auxiliary variable *ω*:

(19) minω

s.t.

(20) $$\omega \geq f_{r}\left(\lambda \right)\;\;\;\;\;\;r=1,\ldots ,s$$

(21) λ∈Ω   

This approach does not require any predetermined preference information from the DM once the objectives and the constraints have been determined, and finally, the answers are presented to it. The DM will not have interfered in the problem-solving process, which is desirable from the point of view of the DM. From Equations (7)–(14), the GDEA_D_ model can be rewritten equivalently as follows:

(22) $$\min\omega_{o}$$

s.t.

(23) $$\overset{n}{\underset{j=1}{\sum }}\left\{\alpha \left(y_{ro}-y_{rj}\right)+{\tilde{d}}_{rj}\right\}\;\lambda_{j}+T\cdot D_{r}^{y}\leq \omega_{o},\;\;r=1,\ldots ,s$$

(24) $$\lambda \in \Omega_{o}\;$$

where $\Omega_{o}=\left\{\lambda :\sum_{j=1}^{n}\left\{\alpha \left(-x_{io}+x_{ij}\right)+{\tilde{d}}_{ij}\right\}\;\lambda_{j}-T\cdot D_{i}^{x}\leq \omega_{o}\;,\forall i;\;\sum_{j=1}^{n}\lambda_{j}=1,\;\lambda_{j}\geq 0,\;\forall j\right\}$.

Certain conditions must be applied to the equivalence between the dual model of GDEA and max-ordering. In Equations (19)–(21), $f_{r}\left(\lambda \right)$ can be written as:

(25) $$f_{r}\left(\lambda \right)=\overset{n}{\underset{j=1}{\sum }}\left\{\alpha \left(y_{ro}-y_{rj}\right)+{\tilde{d}}_{rj}\right\}\;\lambda_{j}+T\cdot D_{r}^{y},\;r=1,\ldots s\;\&\;\;\lambda ={\left(\lambda_{1},\ldots ,\;\lambda_{n}\right)}^{t}$$

Suppose $\Omega_{o}=\Omega$ in Equations (19)–(24), therefore Equations (22)–(24) can be rewritten as:

(26) $$\min\omega_{o}$$

s.t.

(27) $$\omega_{o}\geq \;\overset{n}{\underset{j=1}{\sum }}\left\{\alpha \left(y_{ro}-y_{rj}\right)+{\tilde{d}}_{rj}\right\}\;\lambda_{j}+T\cdot D_{r}^{y}\;\;r=1,\ldots ,s$$

(28) $$\begin{array}{c} \lambda \in \Omega_{o}=\{\lambda :\overset{n}{\underset{j=1}{\sum }}\left\{\alpha \left(-x_{io}+x_{ij}\right)+{\tilde{d}}_{ij}\right\}\;\lambda_{j}-T\cdot D_{i}^{x}\leq \omega_{o},\;i=1,\ldots ,m;\overset{n}{\underset{j=1}{\sum }}\lambda_{j}\\ \;=1,\;\lambda_{j}\geq 0,j=1,\ldots ,n\} \end{array}$$

The relationship between GDEA_D_ and max-ordering can be created by *Theorem 1*:

**Theorem** **1.** *Using formulations in Equations (25)–(30), the GDEA_D_ Equations (22)–(24) replaced with the max-ordering Equations (19)–(21):*

(29) $$\Omega_{o}=\Omega \;$$

(30) $$\omega_{o}=\omega$$

**Proof.**  GDEA_D_ can be rewritten as Equations (31)–(33) using formulations in Equations (25)–(30):

(31) minω

s.t.

(32) λ∈Ω  

(33) $$\omega \geq f_{r}\left(\lambda \right)\;\;\;\;\;\;r=1,\ldots ,s\;$$

The GDEA_D_ can be rewritten as the max-ordering. Since Equations (19)–(21) create an efficient and a specific point of Equations (15) and (16), therefore Equations (22)–(24) also create an efficient and a specific point of Equations (34)–(36):

(34) $$\min\;\left[\overset{n}{\underset{j=1}{\sum }}\left\{\alpha \left(y_{1o}-y_{1j}\right)+{\tilde{d}}_{1j}\right\}\;\lambda_{j}+T\cdot D_{1}^{y},\;\ldots ,\overset{n}{\underset{j=1}{\sum }}\left\{\alpha \left(y_{so}-y_{sj}\right)+{\tilde{d}}_{sj}\right\}\;\lambda_{j}+T\cdot D_{s}^{y}\right]$$

s.t.

(35) $$\overset{n}{\underset{j=1}{\sum }}\left\{\alpha \left(-x_{io}+x_{ij}\right)+{\tilde{d}}_{ij}\right\}\;\lambda_{j}-T\cdot D_{i}^{x}\leq \omega_{o},\;\;\;i=1,\ldots ,m$$

(36) $$\overset{n}{\underset{j=1}{\sum }}\lambda_{j}=1\;\;\;\;\;\;\;\lambda_{j}\geq 0,\;\;j=1,\ldots ,n\;$$

Then, Equations (22)–(24) convert the GDEA model to the MOLP model. This model does not contain value judgments or DM’s preferences for efficiency evaluations. Hence, an interactive approach of a multi-objective programming problem can be applied to consider the DM’s preferences in Equations (22)–(24). □

## 7. The Proposed Interactive GDEA Model

Summarising the proposed method leads to the following three main steps:

*Step* *1—Normalisation*: Since the range of data varies widely, normalisation transfers the range of data to scale the range in [0, 1].
*Step* *2—Efficiency evaluation*: The value of α and T, according to the type of GDEA models, can be represented as follows:
  (a) CCR: sufficiently large and positive α and T ≠ 0,
  (b) BCC: sufficiently large and positive α and T = 0,
  (c) FDH: sufficiently small and positive α and T = 0
  (d) The GDEA efficiency ($\omega_{o}$) of all units is evaluated using the GDEA dual Equations (7)–(14). Then, efficient and inefficient units are presented to DM.
  (e) If the DM is not satisfied with some of the inefficient DMUs, these DMUs will become efficient DMUs using the convex combination ($\lambda_{j}$) obtained from Equations (7)–(14). The values of the inputs and outputs of these DMUs are provided to DM; since DM has not selected the outputs, we go to the next step.
*Step* *3—MPS attainment*: The dual GDEA model converts to the MOLP model for selected inefficient DMU*_o_* by DM Equations (34)–(36). If DM is not satisfied with the obtained output level based on Equations (34)–(36), go to the following section.

The preferences are taken from DM and applied to evaluate the efficiency of the considered unit using the interactive approach, such as the STEM method. This section continues until the MPS achieves the final solution. In each iteration, a convex combination ($\lambda_{j})$ is proposed, and the modified levels of outputs are presented to the DM.

## 8. Interactive STEM Method

The STEM technique can be used in the interactive process of MOLP models for finding the MPS. The STEM technique is based on minimising the Tchebychev distance from the ideal point in the vector space. The parameters and the feasible space can be replaced by the normalised weights based on the DM’s preferences of previous solutions. The STEM technique allows the DM to identify the best solutions in each iteration. It improves some objectives by losing other objectives. The DM must adjust a maximum value for each objective function that can be modified; however, there is no need to determine trade-offs between objectives. In each iteration, the STEM technique, given an answer $x^{h-1}$, the DM should provide objective function preferences to be improved $\left\{f_{i},i\in \left\{1,\ldots ,n\right\}-J^{h}\right\}$. Similarly, the objective functions must be released $\left\{f_{i},i\in J^{h}\right\}$ with maximum values of relaxing $\left\{\Delta f_{i}^{h},i\epsilon J^{h}\right\}$. Equations (37)–(41) can be solved via the DM’s preferences.

(37) minα 

s.t.

(38) $$w_{i}\left(f_{i}^{*}-f_{i}\left(x\right)\right)\leq \alpha ,\;\;\;\;\;\;i\in \left\{1,\ldots ,n\right\}-J^{h}$$

(39) $$f_{j}\left(x\right)\geq f_{j}\left(x^{h-1}\right)-\Delta f_{j}^{h},\;j\epsilon J^{h}\;$$

(40) $$f_{j}\left(x\right)\geq f_{j}\left(x^{h-1}\right),\;j\in \left\{1,\ldots ,n\right\}-J^{h}$$

(41) x∈Ω, α≥0

where $f_{i}^{*}=\underset{x\in \Omega }{\max}f_{i}\left(x\right),\;i=1,\ldots n$ are the best values of objective functions.

## 9. Numerical Example in Hospitals That Provide Stroke Care Services

**Figure 4:**
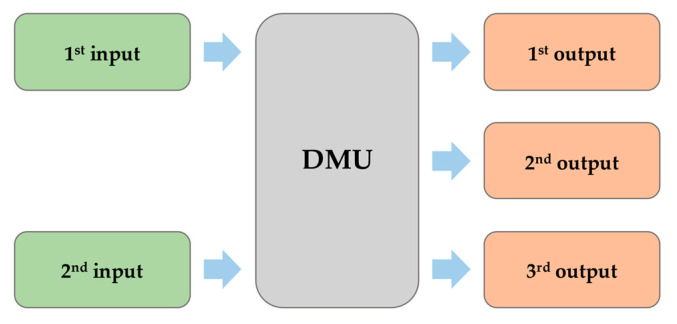
Two inputs and three outputs of the suggested model.

In this section, as a numerical example proposed interactive method for 14 hospital units is considered. Moreover, whether any of these hospitals have a similar number of inputs and outputs in their region is also considered. We use five variables from the data set as inputs and outputs, and Figure 4 shows the two inputs and three outputs derived from the hospital during 2020.

Table 1 shows the description of inputs and outputs in hospitals.

The input variables are an average length of stay (ALOS), average occupational and physical therapy charges (OT/PT), while the output variables are average mild patients, average semi-severe patients, and average severe patients per provider. Table 1 presents inputs and outputs of the proposed model for the stroke care services.

Additionally, Table 2 shows the data set obtained from hospitals in the case study.

The proposed interactive procedures are now implemented in the mentioned problem as follows:

*Step* *1—Normalisation*: The Euclidean norm is used for the normalisation of the data, and the results are presented in Table 3.
*Step* *2—Efficiency evaluation*: According to the FDH model of GDEA, the values of α and T are 1 and 0, respectively. Table 4 presents the efficiency results of the GDEA_D_ Equations (7)–(14) for each unit of the hospital.

As illustrated in Table 4, units 1, 2, 3, 4, 6, 7, 8, and 11 are inefficient units of the hospital. For example, unit 1 has an efficiency value of −0.00521, suggesting that it is an inefficient unit of the hospital. Additionally, Table 4 shows how the input and output values of inefficient units change in the data of efficient units. For example, a convex combination of unit 1 on the efficient frontier can be generated as a linear combination of 0.043 of unit 5, 0.561 of unit 12, and 0.396 of unit 13. Therefore, a convex combination of unit 1 is used to be efficient, and the levels of outputs and inputs are modified as $\left(O_{1},\;O_{2},\;O_{3}\right)=\left(246,\;303,\;296\right)$ and $\left(I_{1},\;I_{2}\right)=\left(165,\;202\right)$.

In fact, the first input (LOS) must be decreased from 168 to 165; the second input (Average OT/PT Charges) must be decreased from 217 to 202. Additionally, the outputs $O_{1}$, $O_{2}$, and $O_{3}$ must be increased to 246, 303, and 296, respectively. However, the hospital’s modified values of inputs and outputs are not accepted as the MPS for unit 1.

*Step 3—MPS attainment*: The modified form of the GDEA dual model as MOLP (with three objects) for DMU 1 (Equations (34)–(36) is as follows:
  (42) $$\begin{array}{c} \min\;[\left\{\left(\left(0.3216-0.3216\right)+{\tilde{d}}_{1,1}\right)\lambda_{1}+\cdots +\left(\left(0.3216-0.2945\right)+{\tilde{d}}_{1,14}\right)\lambda_{14}\right\},\\ \left\{\left(\left(0.217-0.217\right)+{\tilde{d}}_{2,1}\right)\;\lambda_{1}+\cdots +\left(\left(0.217-0.2412\right)+{\tilde{d}}_{2,14}\right)\;\lambda_{14}\right\},\\ \left\{\left(\left(0.3111-0.3111\right)+{\tilde{d}}_{3,1}\right)\;\lambda_{1}+\cdots +\left(\left(0.3111-0.4245\right)+{\tilde{d}}_{3,14}\right)\lambda_{14}\right\}] \end{array}$$
  s.t.
  (43) $$\left\{\left(\left(-0.26+0.26\right)+{\tilde{d}}_{1,1}\right)\lambda_{1}\right\}+\cdots +\left\{\left(\left(-0.26+0.318\right)+{\tilde{d}}_{1,14}\right)\lambda_{14}\right\}\leq \omega_{o}$$
  (44) $$\left\{\left(\left(-0.2624+0.2624\right)+{\tilde{d}}_{2,1}\right)\lambda_{1}\right\}+\cdots +\left\{\left(\left(-0.2624+0.3101\right)+{\tilde{d}}_{2,14}\right)\lambda_{14}\right\}\leq \omega_{o}\;$$
  (45) $$\lambda_{1}+\lambda_{2}+\cdots +\lambda_{14}=1$$
  (46) $$\lambda_{j}\geq 0,\;\;j=1,\ldots ,14\;\;$$
  ${\tilde{d}}_{ij}$ and ${\tilde{d}}_{rj}$ are the components of the matrix $\left(-x_{1o}+x_{1j},\ldots ,-x_{io}+x_{ij}\right)$ and $\left(y_{1o}-y_{1j},\;\ldots ,y_{ro}-y_{rj}\right),\;\mathrm{respectively}$, that is replaced by zero, except for the highest component of vectors. ${\tilde{d}}_{1,1}=$ max (0.3216−0.3216)=0, ${\tilde{d}}_{1,2}=$ max (0.3216−0.1942)=0.1274, …, ${\tilde{d}}_{1,14}=(0.3216-0.2945)=0.0271$. ${\tilde{d}}_{2,1}=\max(0.3216-0.3216,0.217-0.217)=0$, ${\tilde{d}}_{2,2}=\max(0.3216-0.1942,0.217-0.2671)=0.1274$, …, ${\tilde{d}}_{2,14}=\max(0.3216-0.2945,0.217-0.2412)=0.0271$. ${\tilde{d}}_{3,1}=\max(0.3216-0.3216,0.217-0.217,0.3111-0.3111)=0$, ${\tilde{d}}_{3,2}=\max(0.3216-0.1942,0.217-0.2671,0.3111-0.238)=0.1274$, …, ${\tilde{d}}_{3,14}=\max(0.3216-0.2945,0.217-0.2412,0.3111-0.4245)=0.1274$. Additionally, the coefficients of the constraints (${\tilde{d}}_{ij})$ are obtained as ${\tilde{d}}_{1,1}=\max\left(-0.26+0.26\right)=0$, …, $\;{\tilde{d}}_{1,14}=\max\left(-0.26+0.318\right)=0.058$ and ${\tilde{d}}_{2,1}=\max\left(-0.26+0.26,-0.2624+0.2624\right)$, …, ${\tilde{d}}_{2,14}=\max\left(-0.26+0.318,-0.2624+0.3101\right)=0.058$. Then, the MOLP model is as:
  (47) $$\begin{array}{c} \min\;[\left\{0\;\lambda_{1}+0.2548\;\lambda_{2}+\cdots +0.0542\;\lambda_{14}\right\},\;\;\left\{0\;\lambda_{1}+0.0773\;\lambda_{2}+\cdots +0.0029\;\lambda_{14}\right\},\\ \left\{0\;\lambda_{1}+0.2005\;\lambda_{2}+\cdots -0.0863\;\lambda_{14}\}\right] \end{array}$$
  s.t.
  (48) $$0\;\lambda_{1}+\cdots +0.116\;\lambda_{14}\leq \omega_{o}$$
  (49) $$0\;\lambda_{1}+\cdots +0.1057\;\lambda_{14}\leq \omega_{o}\;$$

Now, in order to consider the DM’s preferences in the obtained multi-objective model, the STEM approach can be applied. Applying the STEM method to the obtained model in the first iteration of step 3, the proposed MPS for unit 1 is found as a convex combination ($\lambda_{j})$ of all units as 0.06 of unit 1, 0.071 of 2, 0.074 of 3, 0.066 of 4, 0.08 of 5, 0.05 of 6, 0.155 of 7, 0.066 of 8, 0.045 of 9, 0.105 of 10, 0.059 of 11, 0.06 of 12, 0.057 of 13, and 0.047 of 14. In fact, the output and the input values of unit 1 with the efficiency value of 0.001807 are attained as $\left(I_{1},\;I_{2}\right)=\left(160,\;210\right)$ and $\left(O_{1},\;O_{2},\;O_{3}\right)=\left(178,\;221,\;215\right)$.

The DM is not satisfied with the second objective function by the first iteration and a particular change in outputs is applied, whereas it satisfies the first and third objectives and tends to reduce the first objective by 10 units and the third objective by 40 to improve the second one. In iteration 2, the solution is as a convex combination of 0.018 of unit 1, 0.293 of 2, 0.19 of 3, 0.08 of 4, 0.044 of 5, 0.011 of 6, 0.01 of 7, 0.046 of 8, 0.011 of 9, 0.024 of 10, 0.058 of 11, 0.183 of 12, 0.004 of 13, and 0.022 of 14. The output and the input values of unit 1 with an efficiency value of 0.00161 are obtained as $\left(I_{1},\;I_{2}\right)=\left(164,\;210\right)$ and $\left(O_{1},\;O_{2},\;O_{3}\right)=\left(188,\;240,\;178\right)$.

Again, the DM has not accepted the attained results of iteration 2. This trend has continued until the DM completely is satisfied. The third iteration of the interactive method gives a convex combination of 0.003 of unit 1, 0.014 of 2, 0.095 of 3, 0.004 of 4, 0.025 of 5, 0.002 of 6, 0.007 of 7, 0.006 of 8, 0.001 of 9, 0.005 of 10, 0.422 of 11, 0.394 of 12, 0.011 of 13, and 0.0042 of 14. In fact, the output, and the input values of unit 1 with efficiency value 0.00043 are gained as $\left(I_{1},\;I_{2}\right)=\left(168,\;216\right)$ and $\left(O_{1},\;O_{2},\;O_{3}\right)=\left(220,\;300,\;188\right)$.

After three iterations, the DM accepts the above input and output values. Finally, the MPS has been obtained, and the interactive method has been completed. As can be seen, the efficiency for unit 1 is improved and converted to zero in each iteration. In a similar way, this process can be replicated for all inefficient DMUs 2, 3, 4, 6, 7, 8, and 11. Thus, MPS will be obtained.

By the numerical results, the advantage of the proposed method over the existing approaches has been demonstrated. As can be concluded in the numerical example, in order to find desirable solutions, the process of the proposed method is not complex. The proposed approach provides an MPS, which is the main target of all the interactive methods. Finally, many of the decision-making problems need predetermined preference information. Our independent proposed model does not require predetermined preference information, and this is one of the main advantages of the suggested method, which cannot be seen in recent studies. This method is also applicable in various sectors, such as other healthcare management services as well as energy sector [79,80,81].

## 10. Conclusions and Future Works

A proper medical evaluation and prompt treatment are required to recover from a stroke. According to the American Heart Association and the American Stroke Association, “*time lost is brain lost*.” As a result, innovative techniques and strategies from various disciplines are needed to address the challenges associated with stroke treatment and care services in hospitals and other healthcare facilities. This study presented a GDEA dual model, and the max-ordering approach was used to investigate the relationship between the GDEA dual model and the MOLP in hospitals that provide stroke care services to select the most efficient approach. We showed how to transform the GDEA dual model to MOLP and solve the model interactively. This relationship could provide the base for an interactive method of MOLP for solving the GDEA and locating the MPS in the efficiency frontiers for each DMU. The STEM technique was applied to reflect the DM’s preferences to the GDEA dual model in a combined approach. The supported interactive method is simpler than the former recommended methods and does not require predetermined preference information. A numerical example is offered to display how the novel proposed interactive method can be applied in the decision-making problem. The numerical example showed how the combined approach supported target setting and efficiency in solving the GDEA model and achieved DM’s MPS. In the future, there will be several follow-up research topics. One is to extend the application of the non-radial or non-oriented GDEA model in practice. Another interesting topic for research is to study robust inverse GDEA. This paper provides a complete framework of the interactive GDEA dual model and MOLP, but the data for GDEA may be imprecise in production activities. Hence, imprecise interactive GDEA should be considered. Finally, applying the suggested interactive model will be helpful for other industries, such as gas companies and banking system evaluations.

## Figures and Tables

**Table 1:** The description of inputs and outputs in hospitals.

Descriptions	Inputs/Outputs
Average Length of Stay (ALOS) in hours	Inputs
Average OT/PT Charges in 10$	
Average severe patients	Outputs
Average semi-severe patients	
Average mild patients	

**Table 2:** The data relates to 14 hospitals.

Output 3 (Average Mild Patients)	Output 2 (Average Semi-Severe Patients)	Output 1 (Average Severe Patients)	Input 2 (Average OT/PT Charges)	Input 1 (Average Length of Stay (ALOS))	No. Hospital
291	198	242	217	168	1
176	243	185	177	187	2
133	247	103	278	115	3
133	129	170	266	151	4
171	222	115	219	105	5
340	122	155	291	172	6
176	197	103	203	124	7
201	236	207	188	184	8
240	325	298	131	273	9
214	115	141	119	167	10
185	355	201	249	175	11
195	272	281	167	177	12
451	357	210	249	154	13
291	198	330	257	206	14

**Table 3:** The normalised data of the Hospitals.

Output 3 (Average Mild Patients)	Output 2 (Average Semi-Severe Patients)	Output 1 (Average Severe Patients)	Input 2 (Average OT/PT Charges)	Input 1 (Average Length of Stay (ALOS))	No. Hospital
0.3111	0.217	0.3216	0.2624	0.26	1
0.238	0.2671	0.1942	0.2138	0.2885	2
0.1336	0.2709	0.1478	0.3356	0.1771	3
0.2189	0.1413	0.1475	0.3216	0.2333	4
0.148	0.2439	0.1895	0.2644	0.1622	5
0.1996	0.1337	0.376	0.3511	0.2648	6
0.1325	0.2165	0.1949	0.2458	0.192	7
0.2665	0.2594	0.2219	0.2275	0.2844	8
0.3829	0.3576	0.2657	0.1582	0.4205	9
0.1819	0.1267	0.2372	0.1439	0.2582	10
0.2585	0.3897	0.2049	0.3003	0.2702	11
0.3612	0.2983	0.2157	0.2025	0.2735	12
0.2709	0.3921	0.4986	0.3015	0.2381	13
0.4245	0.2412	0.2945	0.3101	0.318	14

**Table 4:** The convex combination and the efficiency of DMUs.

λ_14_	λ_13_	λ_12_	λ_11_	λ_10_	λ_9_	λ_8_	λ_7_	λ_6_	λ_5_	λ_4_	λ_3_	λ_2_	λ_1_	$\omega_{o}$	
	0.396	0.561							0.043					−0.00521	1
		0.941		0.054	0.001									−0.01463	2
	0.186								0.814					−0006.73	3
		0.437							0.563					−0.02238	4
									1					0	5
	0.744	0.019							0.237					−0.0439	6
	0.002	0.105		0.139					0.754					−0.0046	7
	0.087	0.793		0.085					0.035					−0.01923	8
					1									0	9
				1										0	10
	0.98				0.02									−0.00172	11
		1												0	12
	1													0	13
1														0	14

## Data Availability

The data used in the study is available with the authors and can be shared upon reasonable requests.
